# Comparison of adverse events in partial- or whole breast radiotherapy: investigation of cosmesis, toxicities and quality of life in a meta-analysis of randomized trials

**DOI:** 10.1186/s13014-023-02365-7

**Published:** 2023-11-02

**Authors:** Jan Haussmann, Wilfried Budach, Stefanie Corradini, David Krug, Danny Jazmati, Bálint Tamaskovics, Edwin Bölke, Alessia Pedotoa, Kai Kammers, Christiane Matuschek

**Affiliations:** 1 Department of Radiation Oncology, Medical Faculty and University Hospital Düsseldorf, Heinrich-Heine-University Düsseldorf, Moorenstr. 5, 40225 Düsseldorf, Germany; 2 Department of Radiation Oncology, Ludwig Maximillian University, Munich, Germany; 3 Department of Radiation Oncology, University Hospital Schleswig-Holstein, Kiel, Germany; 4 Department of Anesthesiology, Memorial Sloan Kettering Cancer Center, New York City, New York USA; 5 Department of Oncology, The Sidney Kimmel Comprehensive Cancer Center, The Johns Hopkins University School of Medicine, Baltimore, USA

**Keywords:** Meta-analysis, Breast cancer, Radiotherapy, Partial breast irradiation, Quality of life, Side effects, Cosmesis

## Abstract

**Purpose/objective:**

Adjuvant whole breast radiotherapy and systemic therapy are part of the current evidence-based treatment protocols for early breast cancer, after breast-conserving surgery. Numerous randomized trials have investigated the therapeutic effects of partial breast irradiation (PBI) compared to whole breast irradiation (WBI), limiting the treated breast tissue. These trials were designed to achieve equal control of the disease with possible reduction in adverse events, improvements in cosmesis and quality of life (QoL). In this meta-analysis, we aimed to investigate the differences between PBI and WBI in side effects and QoL.

**Material/methods:**

We performed a systematic literature review searching for randomized trials comparing WBI and PBI in early-stage breast cancer with publication dates after 2009. The meta-analysis was performed using the published event rates and the effect-sizes for available acute and late adverse events. Additionally, we evaluated cosmetic outcomes as well as general and breast-specific QoL using the EORTC QLQ-C30 and QLQ-BR23 questionnaires.

**Results:**

Sixteen studies were identified (n = 19,085 patients). PBI was associated with a lower prevalence in any grade 1 + acute toxicity and grade 2 + skin toxicity (OR = 0.12; 95% CI 0.09–0.18; *p* < 0.001); (OR = 0.16; 95% CI 0.07–0.41; *p* < 0.001). There was neither a significant difference in late adverse events between the two treatments, nor in any unfavorable cosmetic outcomes, rated by either medical professionals or patients. PBI-technique using EBRT with twice-daily fractionation schedules resulted in worse cosmesis rated by patients (n = 3215; OR = 2.08; 95% CI 1.22–3.54; *p* = 0.007) compared to WBI. Maximum once-daily EBRT schedules (n = 2071; OR = 0.60; 95% CI 0.45–0.79; *p* < 0.001) and IORT (*p* = 0.042) resulted in better cosmetic results grade by medical professionals. Functional- and symptom-based QoL in the C30-scale was not different between PBI and WBI. Breast-specific QoL was superior after PBI in the subdomains of “systemic therapy side effects” as well as “breast-” and “arm symptoms”.

**Conclusion:**

The analysis of multiple randomized trials demonstrate a superiority of PBI in acute toxicity as well breast-specific quality of life, when compared with WBI. Overall, late toxicities and cosmetic results were similar. PBI-technique with a fractionation of twice-daily schedules resulted in worse cosmesis rated by patients.

## Introduction

International guidelines recommend breast-conserving surgery (BCS), whole breast irradiation (WBI) and systemic therapy including endocrine therapy, HER2-targeted- and chemotherapy as the appropriate treatment for early-stage breast cancer. This multimodal approach has been shown to be equivalent to mastectomy in numerous randomized trials [1, 2]. Breast conserving surgery also seems to be associated with an improved quality of life (QoL) [3–8].

Scientific and structural advances including diagnostic imaging, high quality pathological testing, less invasive and morbid resection and effective systemic treatment have achieved very favorable oncologic results for most patients with early-stage disease. Women with localized breast cancer have a 99% chance of being alive after 5 years [9].

Several attempts have been put forward to de-escalate the treatment of early-stage breast cancer. Omission of adjuvant whole breast irradiation was studied in multiple randomized trials [10–17]. Published meta-analyses established that forgoing WBI does not impact overall survival in selected patients but is associated with a significantly higher rate of local recurrence [18–20]. Partial breast Irradiation (PBI) was suggested as a technique that limits radiotherapy to the tissue adjacent to the tumor bed which is the most frequent site of local recurrence. Studies of PBI investigated non-inferiority of local control, comparable or better cosmetic results and lower toxicities when compared to WBI. Additionally, PBI was designed to be delivered for shorter treatment durations, being more convenient for patients and less expensive for health care providers.

This analysis will review and analyze the current data on adverse events comparing WBI to PBI, their side effects and cosmesis. The results on survival as well as local control rates were published separately [21, 22].

## Material and methods

On March 1st, 2023 we performed a literature review according to the published PRISMA guideline [23]. We searched the PubMed library with the search terms (“radiation therapy” or “radiotherapy” or “irradiation”) AND ("breast cancer" or "carcinoma of the breast") AND ("partial" or "targeted") AND (“randomized” OR “randomized” OR “randomly”). In addition, we screened the major meetings (e.g. ASTRO-, ESTRO-, ESMO-, ASCO annual meetings) for published abstracts. The search was supplemented by hand searches. We included randomized controlled trials including patients suffering from early-stage invasive breast cancer comparing PBI to WBI with a language restriction to English. Trials had to be published after the year 2009 in order allow for comparable modern techniques.

### Selection of endpoints

All available toxicity and QoL analyses were retrieved from the published literature and filtered for matching scales and follow-up time. In order to include the highest possible number of trials we chose the endpoint and scoring used in the majority of trials. In the toxicity and QoL analyses, the longest available follow-up time was used to insure adequate capture of adverse events. In the evaluation of cosmetic events, we stratified by follow-up time. In order to compare different PBI techniques and external beam radiation therapy (EBRT) fractionation schedules, we performed an analysis using all available data that included non-standard protocol-specific endpoints. We hierarchically used the grade 2 + toxicities, or when unavailable, grade 3 + and subsequently grade 1 + toxicities.

### Toxicities

Acute and late toxicities were generally reported on the EORTC/RTOG or LENT-SOMA scales which are 6 and 5 point Likert-scales (0–4/5) in increments of 1 with the main difference of death from toxicity (5) only scorable in the EORTC/RTOG scoring system. We also included other interval scales like the four point used in the IMPORT LOW-trial reporting dichotomized when we were investigating relative effect sizes in the study arms.

The cosmetic results were obtained at the latest available time point according to a four point scale (excellent/good/fair/poor) in most trials [24–34]. The cosmetic assessment by the physicians from the IMPORT LOW trial was based on the photographic assessment in a three-point scale (no change [none], mild, or marked change) and reported for the mild and marked change at the five year timepoint [35]. Cosmetic results evaluated by the patients were scored in 4-point scale. The item “Breast appearance changed” was dichotomized in the 4-point scale (not at all, a little, quite a bit, or very much) and used from the IMPORT LOW trial at the five years follow-up timepoint. The DBCG PBI trial assessed patient rated cosmesis in the item “Patient satisfaction, compared with contralateral breast” on a 4-point scale [36]. When the objective assessment included both physicians and nurses, we used the evaluation of the trained nurses as the might provide a more unbiased assessment [26, 34].

### Quality of life analysis

Three trials used the EORTC QLQ-C30 questionnaire for general quality of life and the QLQ-BR23 questionnaire for breast- cancer specific quality of life. Both scales are subdivided into functional and symptomatic scales.

The IMPORT LOW trials also assessed QoL with the QLQ-BR23 questionnaire and protocol specific questions. However, the published analysis was restricted to the dichotomized values for moderate or marked responses. The NSABP B-39 trial reported several patient assessments including QoL measured by the BCTOS scale. Due to these differences, we were unable to include both trials in the assessment. The predefined threshold for minimal clinically meaningful difference was set at values of 5 or above [37].

### Endpoints

We extracted the provided hazard ratios, odds ratios or event numbers from the identified trials to estimate the effect sizes comparing WBI to PBI in the endpoints of acute adverse events including any acute toxicities, acute skin toxicity, pneumonitis and breast pain. Late adverse events included any late toxicities, late skin toxicity, late subcutaneous/fibrosis/induration, telangiectasia, breast pain, chest wall pain, breast edema, fat necrosis and pulmonary toxicity. Bone toxicity according to RTOG scale was pooled with chest wall pain from the Common Terminology Criteria for Adverse Events (CTCAE) Version 3.0 [26, 33].

### Subgroups

In order to compare different techniques, we grouped the trials according to PBI technique into external beam radiation (QD = a maximum of once daily treatment and BID = twice daily therapy), intraoperative radiotherapy using electrons or photons and any interstitial or applicator-based brachytherapy. If a trial included more than one technique, the trials were reported as the combination of the two techniques, except when separate data were available. The respective PBI techniques in Figs. 2, 4, 5, 6 and 7 are: mixed (green), EBRT BID (light blue), darker blue (EBRT QD), red (IORT), orange (BT).

### Statistics

The comparison of acute and late adverse events and cosmetic results was obtained using odds ratios. QoL subcategories from the EORTC QLQ-C30 and EORTC QLQ-BR23 scales were compared with weighted mean differences (WMD). We used the inverse variance heterogeneity model (ivhet) to pool effect sizes, as this model uses a more conservative estimate of the confidence limits, has less observed variances and favors larger trials compared to the commonly used random effects model [38]. Zero event correction was applied where appropriate [39]. Statistical significance limit was set at p-values lower than 0.05. Significant values are marked in bold letters for better visibility. Heterogeneity within the meta-analysis was obtained with Cochran’s Q-test with the corresponding *p* values. The I^2^ statistics were also described, with values above 25% identified as considerable heterogeneity, triggering a subgroup analysis by technique [40]. Funnel plots were created to assess publication bias. For statistical analysis, Microsoft Excel add-in MetaXL 5.3 was used (EpiGear International, Sunrise Beach, Australia). Plots were created using Microsoft Excel for Microsoft Office 365 Pro Plus (Redmond, Washington, U.S.).

## Results

The literature analysis (Appendix Fig. 8) identified 51 publications reporting 16 different randomized trials. These included an overall number of 19,085 patients. Median follow-up for the primary endpoints was 8.6 years. Appendix Tables 1 and 2 show an overview of the included trials with important patient characteristics, treatment details and toxicity endpoints.

### Acute toxicity

**Fig. 1:**
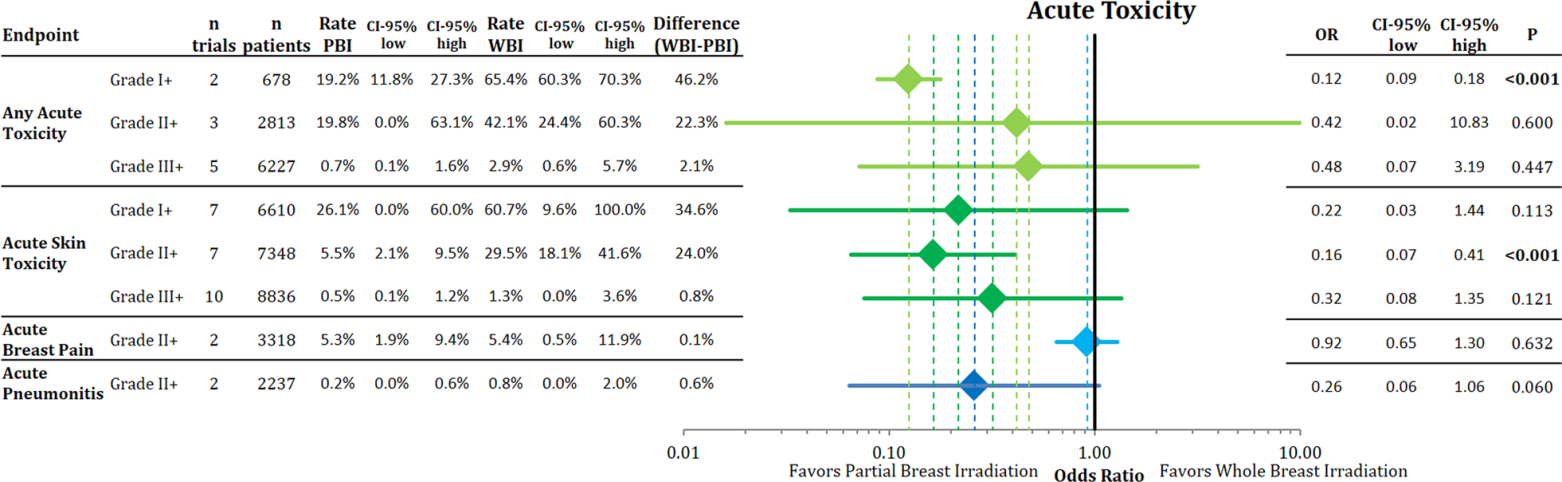
Comparison of acute toxicities sorted by grade using pooled rates and odds ratio with the respective 95% confidence intervals between partial breast radiotherapy and whole breast radiotherapy

The comparison in acute toxicities between PBI and WBI is described in Fig. 1. PBI was associated with a significant decrease in acute adverse events equal or higher than grade I (n = 678; OR = 0.12; 95% CI 0.09–0.18; *p* < 0.001) and a reduction in acute skin toxicity, specifically grade II + radiodermatitis (n = 7348; OR = 0.16; 95% CI 0.07–0.41; *p* < 0.001). Grade II + acute skin toxicity occurred in 5.5% (95% CI 2.1–9.5%) of patients treated with PBI compared to 29.5% (95% CI 18.1–41.6%) with WBI. There were no statistically significant differences regarding grade II + acute pneumonitis (OR = 0.26; 95% CI 0.06–1.06; *p* = 0.060), or grade II + breast pain (OR = 0.92; 95% CI 0.65–1.30; *p* = 0.632) between the two modalities. In addition to the shown endpoints, PBI decreased acute breast edema, as analyzed in the RAPID trial (n = 2135; OR = 0.68; 95% CI 0.49–0.95; *p* = **0.023**). Acute fatigue was not statistically different between the two groups (n = 2135; OR = 0.90; 95% CI 0.71–1.16; *p* = 0.423).

**Fig. 2:**
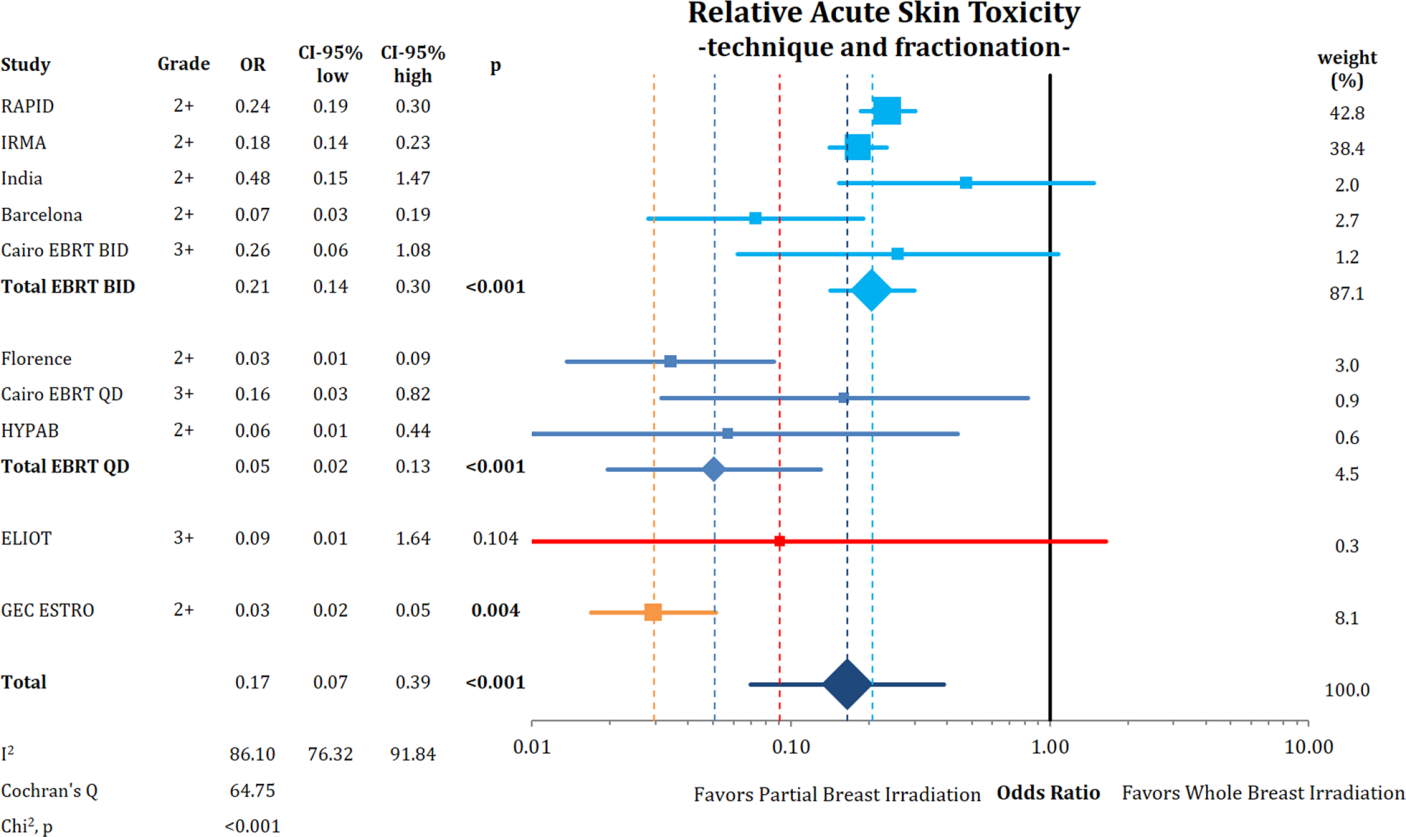
Comparison of acute skin toxicity using odds ratios with the respective 95% confidence intervals between partial breast radiotherapy and whole breast radiotherapy according to technique and fractionation schedule. The red and orange lines indicate PBI with IORT and BT, respectively

The analysis of relative acute skin toxicity by subgroup is shown in Fig. 2. A relative reduction of acute toxicity was shown for all PBI methods, except for IORT (*p* = 0.104).

### Late toxicity

**Fig. 3:**
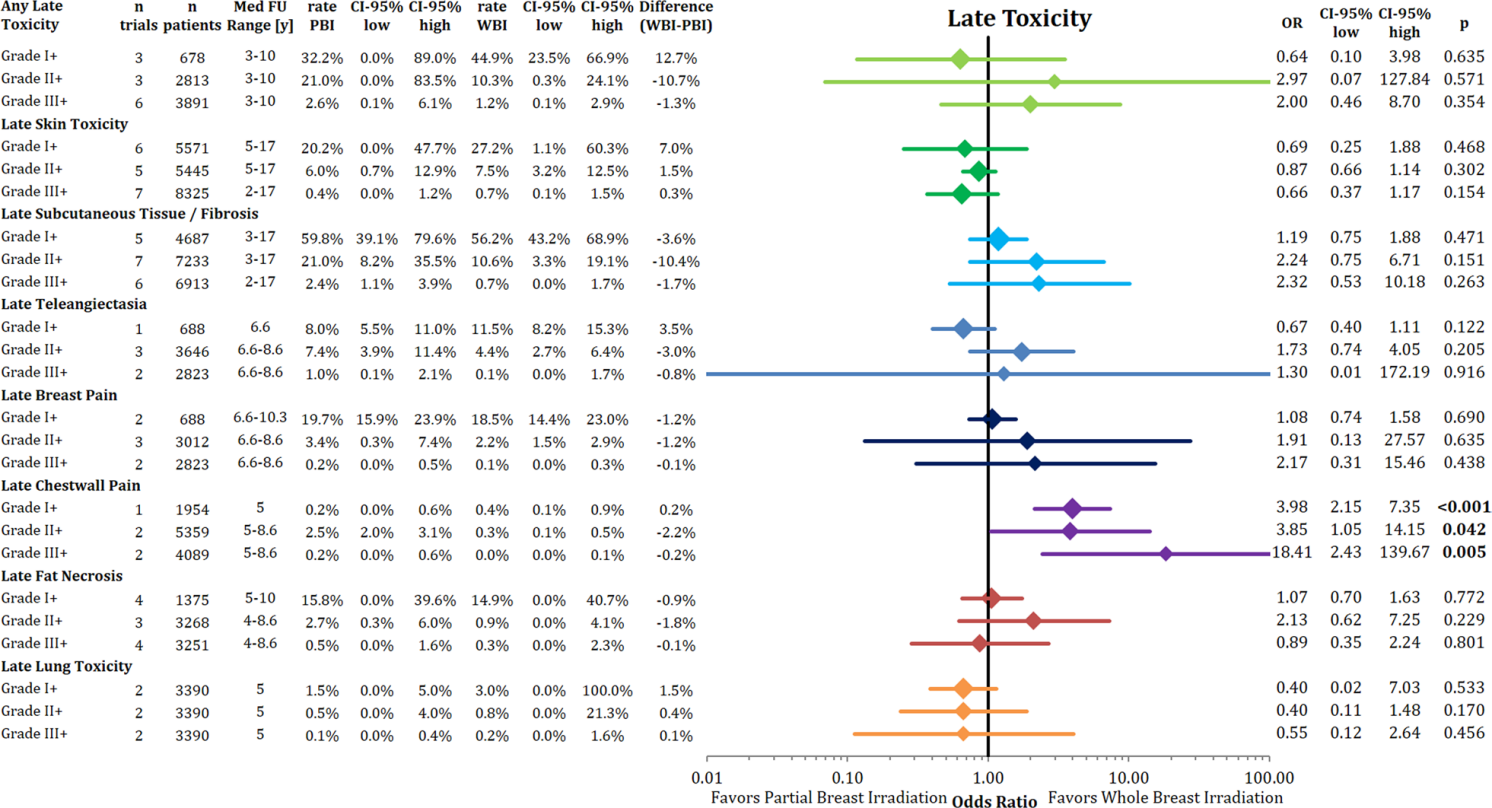
Comparison of late toxicities sorted by grade using pooled rates and odds ratio with the respective 95% confidence intervals between partial breast radiotherapy and whole breast radiotherapy

The combined analysis of all assessed late toxicity endpoints separated by grading is presented in Fig. 3. Any late toxicity, late skin toxicity, late subcutaneous fibrosis/induration, late telangiectasia, late breast pain, fat necrosis and lung toxicity were not different between PBI and WBI as shown in Fig. 3. PBI resulted in more late chest wall pain in all analyzed grades.

When the PBI technique was analyzed separately (Appendix Fig. 9), there was no difference in any late adverse events between the trial groups (OR = 2.05’; 95% CI 0.45–9.39; *p* = 0.357). Numerically, EBRT BID had higher rates of late adverse events (OR = 2.05; 95% CI 0.39–24.13; *p* = 0.285), but without reaching the threshold of statistical significance. There was also no difference in late skin adverse events like fibrosis, by either radiation modality or technique (Appendix Fig. 10). In the sub-analysis of late subcutaneous tissue toxicity by different radiation techniques, the overall comparison likewise did not detect a significant difference (OR = 2.00; 95% CI 0.89–4.51; *p* = 0.094) (Appendix Fig. 11) Patients treated with brachytherapy suffered more subcutaneous tissue toxicity (OR = 1.66; 95% CI 1.03–2.67; *p* = 0.037).

### Cosmesis

**Fig. 4:**
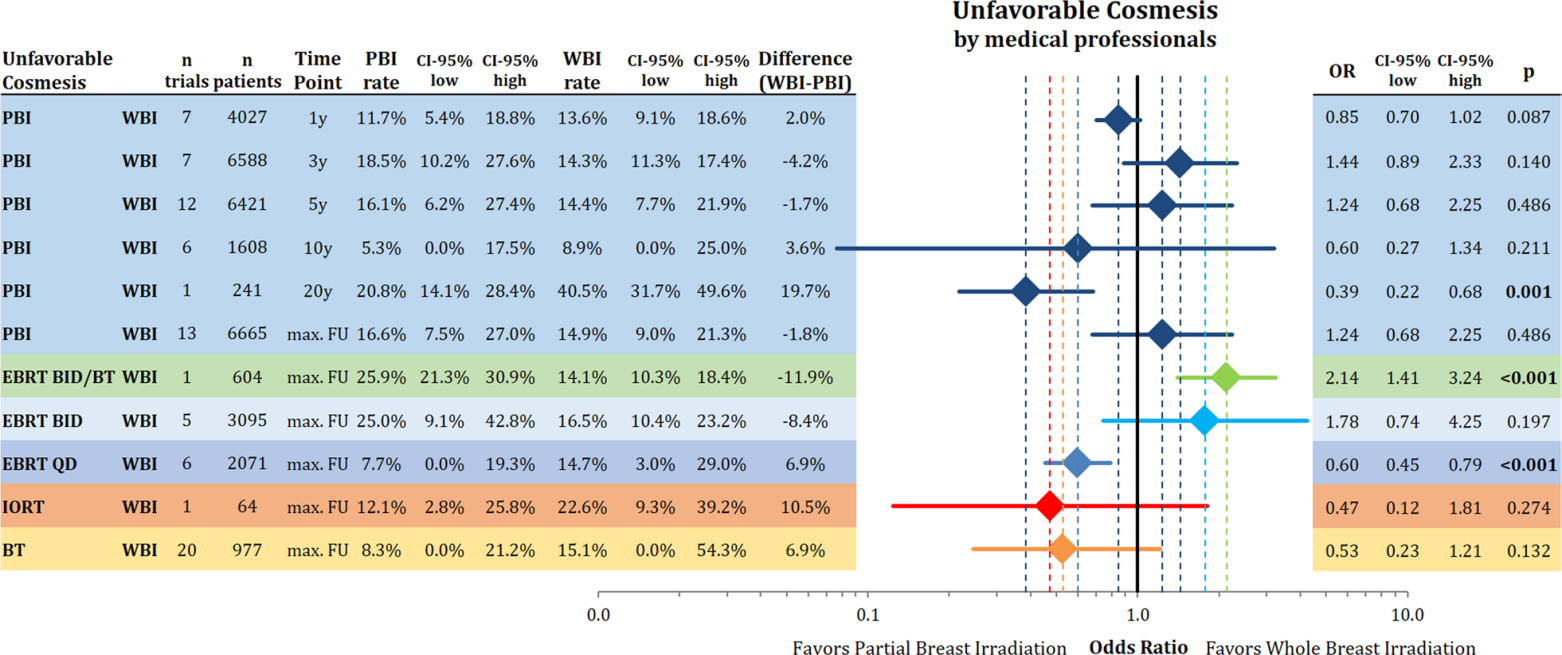
Comparison of unfavorable cosmesis (fair/poor) rated by medical professionals by different time points and technique using odds ratios with the respective 95% confidence intervals between partial breast radiotherapy and whole breast radiotherapy

Unfavorable cosmetic outcome (fair or poor on the 4-point scale) rated by medical professionals (physicians or nurses) was not different between the treatments after 1, 3, 5, 10 years and maximal follow-up, but reached statistical significance at the 20th year time point. However, this was based on data from a single trial (Fig. 4). The analysis by technique using all available maximal follow-up time intervals showed a significantly worse cosmesis for EBRT BID/BT (n = 604; rate PBI: 25.9%; rate WBI: 30.9%; OR = 2.14; 95% CI 1.41–3.24; *p* < 0.001), while once a day EBRT resulted in less cosmetic deterioration (n = 2071; rate PBI: 7.7%; rate WBI: 14.7%; OR = 0.60; 95% CI 0.45–0.79; *p* < 0.001). There was a trend towards worse cosmesis using the point estimates for EBRT BID, while numerically BT and IORT were associated with improved cosmesis.

**Fig. 5:**
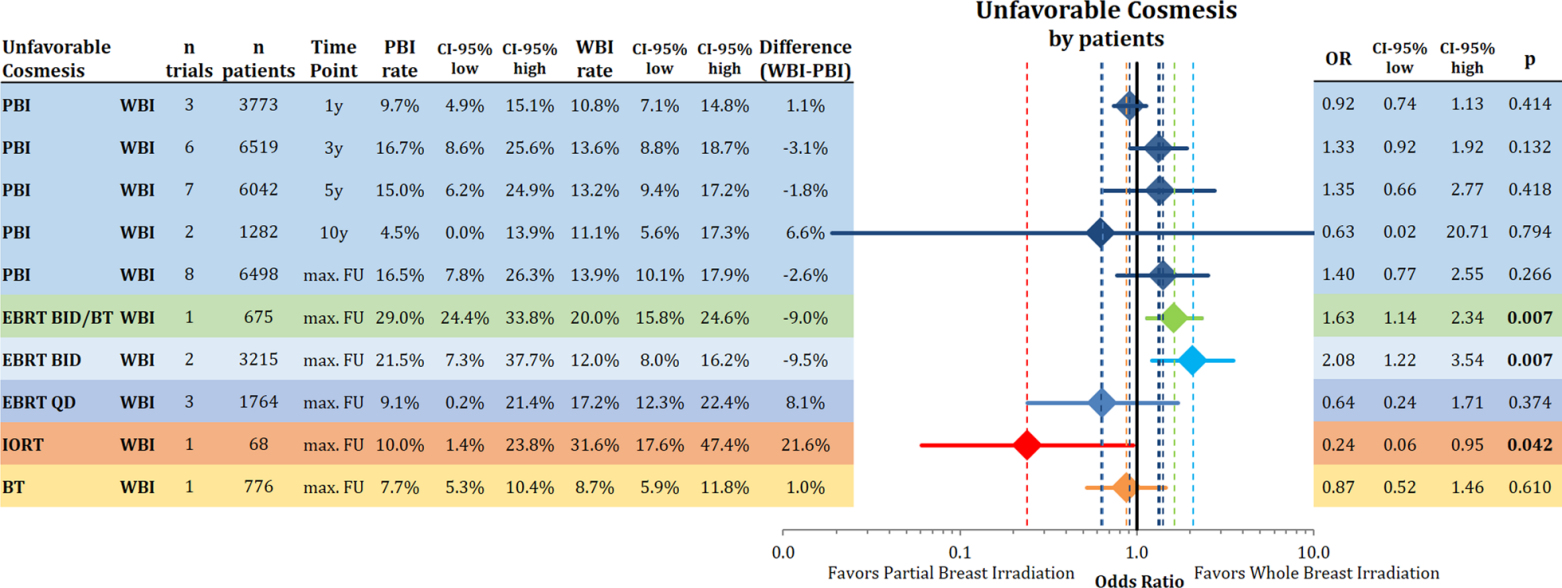
Comparison of unfavorable cosmesis (fair/poor) rated by patients by different time points and technique using odds ratios with the respective 95% confidence intervals between partial breast radiotherapy and whole breast radiotherapy

When using the patients’ assessment of breast cosmesis we obtained similar results. Overall, unfavorable cosmesis was not different between PBI and WBI at the timepoints 1y, 3y, 5y, 10y and maximal follow-up. Both EBRT BID/BT (n = 675; OR = 1.63; 95% CI 1.14–2.34; *p* = 0.007) and EBRT BID (n = 3215; OR = 2.08; 95% CI 1.22–3.54; *p* = 0.007) resulted in significantly worse cosmetic results. Patients receiving IORT reported better results (n = 68; OR = 0.24; 95% CI 0.06–0.95; *p* = 0.042) (Fig. 5).

### QoL assessment

Quality of life was scored by the EORTC QLQ-C30 and QLQ-BR23 questionnaires divided into functional and symptom scales. The pooled QLQ-C30 functional items (global, physical function, role function, emotional function, cognitive function and social function) did not show any differences between PBI and WBI (Appendix Fig. 12). Notably, all comparisons had significant heterogeneity with superior QoL in all items in the assessment of the Florence trial.

**Fig. 6:**
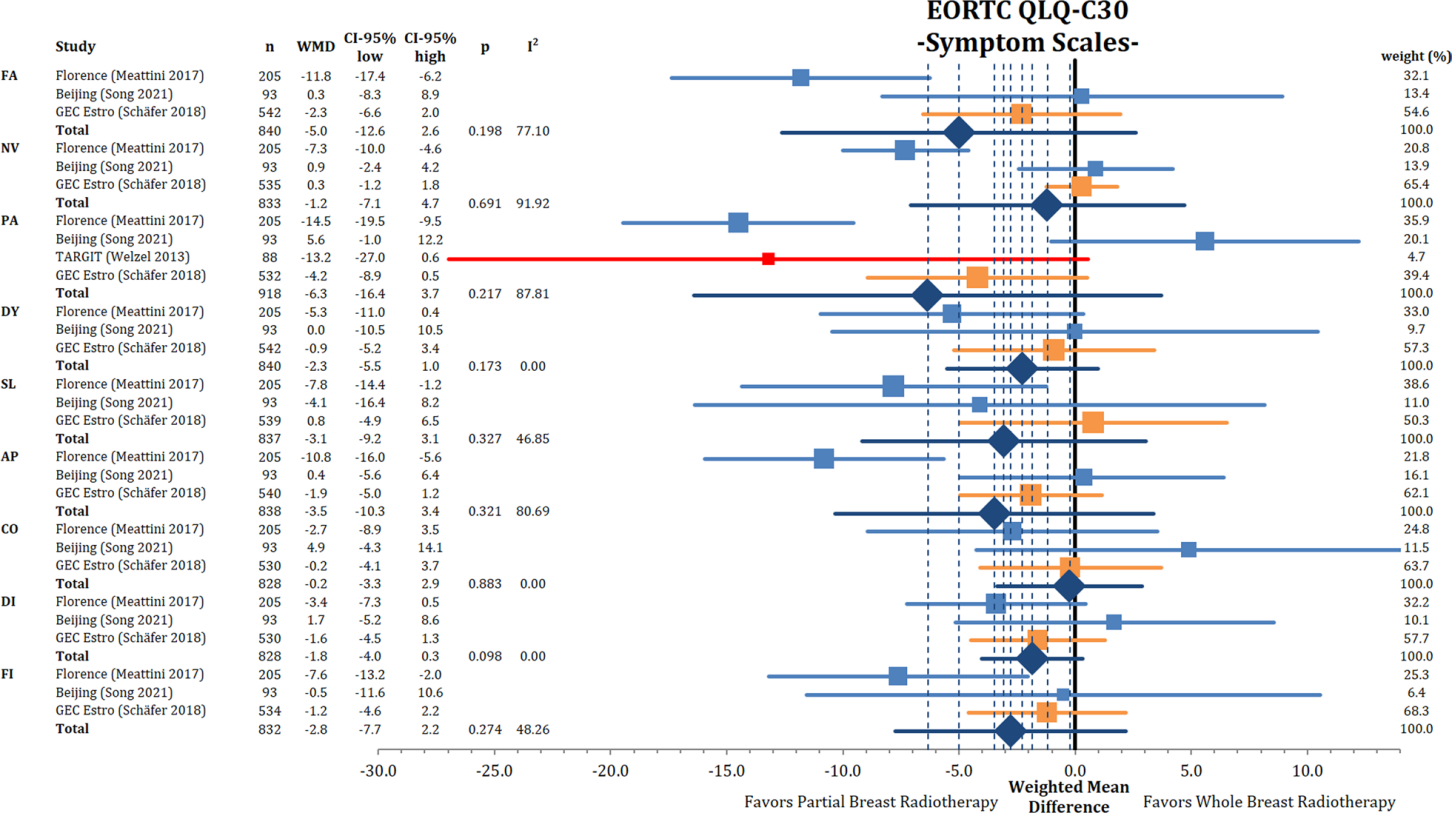
Comparison of QLQ-C30 scores between partial breast irradiation and whole breast irradiation in different subdomains using weighted mean differences. Lower symptom scores represent better QoL. FA = fatigue, NV = nausea and vomiting, PA = pain, DY = dyspnea, SL = insomnia, AP = appetite loss, CO = constipation, DI = diarrhea, FI = financial difficulties

The analysis of the QLQ-C30 symptom scales (Fig. 6) did not show a significant improvement in any subscale when analyzing all PBI techniques together. Numerically, all point estimates were superior in the PBI arm. Clinically meaningful differences are reported for the PBI arm in the Florence trial for fatigue, pain and appetite loss.

The effect of PBI versus WBI on breast-specific quality of life was compared using the EORTC QLQ-BR23. The functional scales on body image, sexual functioning, sexual enjoyment and future perspective were not significantly different between PBI and WBI (Appendix Fig. 13). CMDs were present in the PBI arm of the Florence trial in the items body image, sexual enjoyment, and future perspective.

**Fig. 7:**
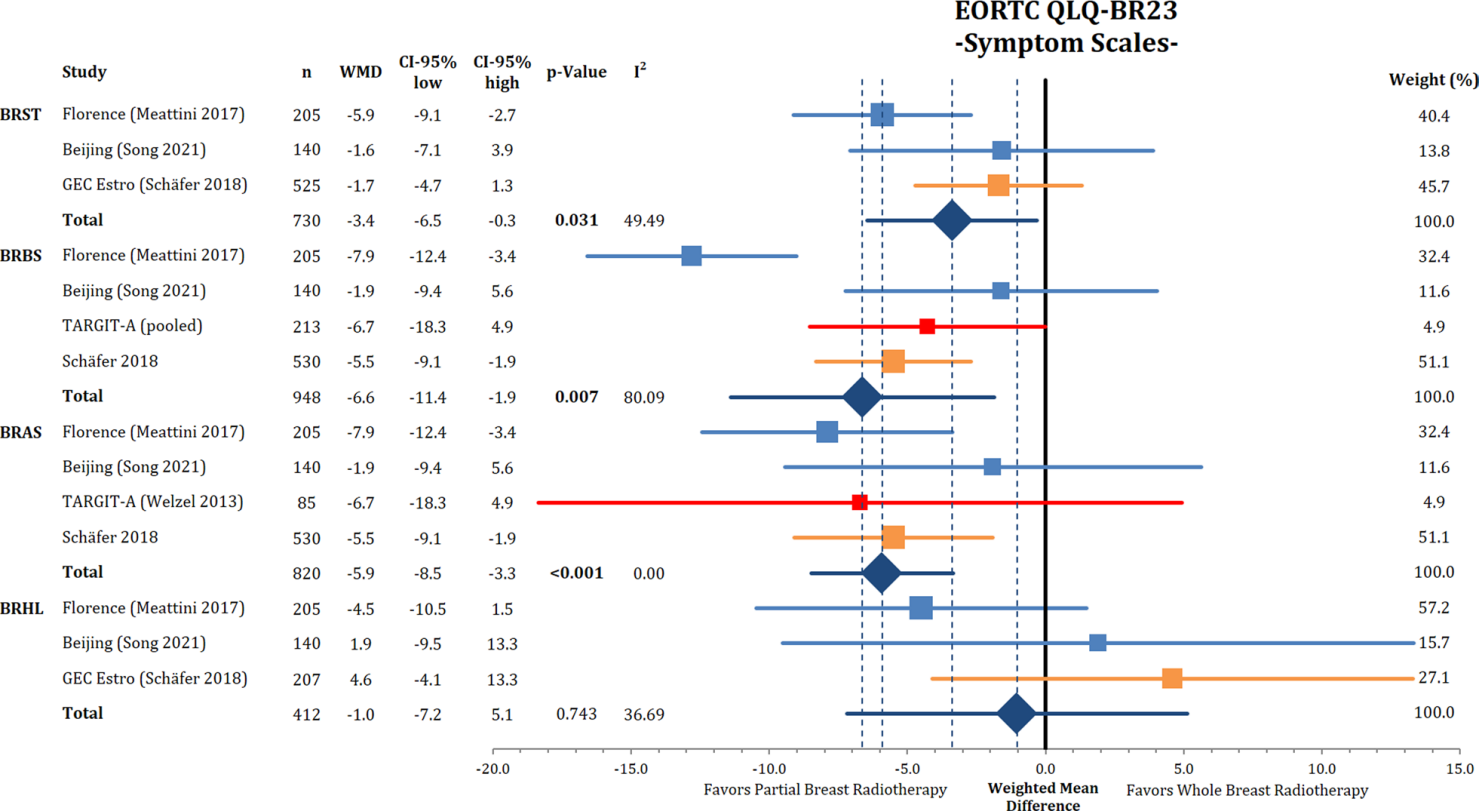
Comparison of QLQ-BR23 scores between partial breast irradiation and whole breast irradiation in different subdomains using weighted mean differences. Lower symptom scores represent better QoL. BRST = systemic therapy side effects, BRBS = breast symptoms, BRAS = arm symptoms, BRHL = hair loss

The analysis of the EORTC QLQ-BR23 symptom domains showed that patients receiving PBI reported significantly fewer systemic side effects, breast and arm symptoms (BRST: WMD = − 3.4; 95% CI − 6.5 to 0.3; *p* = 0.031) (BRBS: WMD = − 6.6; 95% CI − 11.4 to 1.9; *p* = 0.007); (BRAS: WMD = − 5.9; 95% CI − 8.5 to 3.3; *p* < 0.001) (Fig. 7). Notably, the pooled differences did not exceed the threshold for CMD.

## Discussion

This meta-analysis compares the side effects of partial and whole breast radiotherapy. While PBI seems to be associated with less acute toxicity and better breast-specific QoL, the effects on late and cosmetic events are similar to whole breast radiation. However, when analyzing the pooled estimates, it is important to consider that the radiation fractionation schedule, influenced the late adverse events as well as the cosmetic breast appearance.

The reduction in treated breast volume led to a noticeable relative decrease in acute skin toxicity by 83%. With an estimation of grade 2+ acute skin toxicity of around 29.5% in WBI and 5.5% in PBI, this difference should be clinically meaningful and might be considered by the treating physicians. However, PBI did not result in a reduction in grade 3 skin toxicity which occurred in less than 4% of patients.

Acute side effects appear to be heavily influenced by the treated volume while PBI reported a lower incidence of acute skin reactions of well as strong tendencies in pneumonitis and any acute side effects. The lack of statistical significance in some of the acute toxicity endpoints might be explained by the conservative comparative model used in this investigation. When the alternatives of fixed effect or random effect models were used, the results showed significant differences between the two treatment modalities. The improvement in acute toxicities was similar in all used radiation techniques and was consistent with the reported prospective and retrospective data as well as the published systematic reviews [41–48]. Therefore, different PBI procedures did not seem to have a relevant impact on acute adverse events as all point estimates favored PBI.

The assessment of late adverse events and cosmetic outcomes showed no overall significant differences. Unfavorable cosmetic results were detected after PBI in about 17% by both medical professionals and patients whereas WBI resulted in impaired cosmesis in 15% and 14% respectively. However, significant heterogeneity in the comparison suggested an association with the radiation technique and the fractionation schedules used. Partial breast treatment with once daily EBRT, BT and IORT might be associated with improved cosmesis. The analyses showed a consistent harmful effect of twice-daily external beam radiotherapy in any late adverse outcome measures and cosmesis as previously hypothesized [49]. Five trials used twice daily radiation schedules [24, 26, 30, 50, 51]. A proposed explanation for this observation is an insufficient normal tissue recovery time between fractions, which was initially anticipated to be less than 6 h. Other authors suggested that an inhomogeneous dose distribution with excessive hotspots could contribute to this finding. However, the published trial protocols did not allow non-standard radiation dose maxima in the target volume. Moreover, other techniques of BT and KV-based IORT also apply inhomogeneous doses and reported favorable cosmesis.

Younger age, larger breast size/surgical deficit, lymph node positivity and higher levels of anxiety/depression have been reported as adverse outcome predictors [52], while previous breast infection/surgical complication, seroma, higher age, smoking status, larger breast volume, greater volume of breast excised, central or inner tumor location, application of a tumor bed boost and use of a conventionally fractionated treatment schedule for whole breast irradiation [53–57] are predictors of poor cosmetic results.

Patients receiving a radiation boost of the tumor bed have higher incidence of tissue fibrosis. The use of a boost doubled the cumulative incidence of moderate or severe fibrosis from 15 to 30% after 20 years of follow-up [55] and it was likely a contributing factor for induration when PBI and WBI were compared. The criteria and frequency of the treatment in the included trials were not standardized, therefore any imbalance in these risk factors could contribute to the obtained results, even though unlikely, due to the randomized distribution of patients to the treatment arms.

Numerous publications have investigated the effects of different radiation schedules on the volume of breast fibrosis and cosmesis [58, 59]. Peterson and colleagues analysed predictors of poor cosmesis in the RAPID trial [53]. Their multivariable model did not demonstrate a significant impact of high dose treatment volume on adverse cosmesis. A detailed analysis from the DBCG-PBI trial that used 40 Gy in 15 fractions in both treatment arms demonstrated that the volume of breast treated with 40 Gy (V40Gy) was closely linked to a diagnosis of breast induration. This observation would support the use of PBI in larger breast sizes. Recently, the DBCG group showed that this correlation is true for women that are older than 65 years (Thomsen et al. ESTRO 2023, Vienna).

A substudy of the TARGIT-A trial focusing on adverse events with the use of IORT, reported better cosmetic results and less acute and late side effects. However, this was a single center outcome analysis within the trial, limiting a widespread applicability of their results. Further, the differences in the experimental arm between patients receiving TARGIT alone and TARGIT with additional whole breast radiation raise concerns regarding the long-term effects of the combined treatment approach [60].

Biologically effective doses (BED) used in PBI arms of the included trials differed significantly. Assuming an alpha/beta ratio of 4 the PBI techniques delivered the following dose ranges: EBRT QD (66.8–75.6 Gy), EBRT BID (62.9–75.6 Gy), IORT (120–131.2 Gy) and BT (62.9–83.7 Gy). The observed changes in the cosmetic outcome between the two EBRT techniques albeit almost no differences in BEDs as well as no relevant differences in the IORT arms despite much higher BEDs given suggest that other factors are mainly contributing to these findings.

The detectable impact of the addition of whole breast radiotherapy compared to endocrine therapy alone on QoL appears to be small. In the PRIME I trial only “breast symptoms” were more pronounced in the radiotherapy arm and resolved after 3 years [11]. This was confirmed by another prospective assessment of QoL during radiotherapy compared to no WBI [61]. Age, socioeconomic status of the patient, administration of chemotherapy or endocrine therapy, BMI and higher baseline anxiety scores are well known factors associated with poor QoL [7, 62–66]. Randomization should help decrease bias related to group allocation. With a threshold of 5 points for clinically meaningful difference, we detected improved QoL only in the Florence study, using EBRT with IMRT and treatment in QD schedules. Other trials and the pooled results did not reach a statistically significant threshold. The analysis was limited by the number of trials and patients included to detect smaller differences. Moreover, pooling of the results might be influenced by the different follow-up times of the trials, as a phenomenon called the “response shift” could influence the QoL scores explained by an adaption of the individual’s QoL assessment [67, 68]. However, other studies have already demonstrated that even a numerical small change in QoL scores could result in a clinically meaningful difference for the individual patient [69].

Our results are consistent with a prospective evaluation of different cohorts receiving IORT, PBI (EBRT QD) and various WBI regimes showed small differences in QoL. Breast symptoms were better after IORT and EBRT compared to WBI, in addition to decreased fatigue, global health status and role functioning over time. These differences were limited to a 2 years-period.

The comparison to other published meta-analyses is difficult because of different data pooling methods, outcome measures and selected endpoints [47, 48, 70, 71]. The scientific evidence for partial breast radiotherapy is also supplemented by numerous prospective single arm trials which also endorsed the same approach as in the trials included in our paper. BID EBRT with total doses of 38 Gy or above seemed to provide excellent/good cosmesis in less than 90% and a substantial rate of induration or fibrosis [59, 72–75]. When the total dose was reduced to 34–38 Gy, the treatment appeared to be better tolerated [42, 76]. It is possible that a reduction in single fraction and overall dose in the BID EBRT treatment might reduce breast induration and mitigate the observed higher toxicity rates. In the small trial from India included in this analysis, this approach showed early favorable results [51].

The other analyzed PBI techniques, the trials using once daily EBRT trials reported excellent/good cosmesis above 88% [41, 44, 45, 76, 77]. Brachytherapy using “Mammosite” also described good cosmetic results (excellent/good above 90%) [78, 79], whereas results with interstitial brachytherapy caused more diverse results (excellent/good: 68–94%) [43, 46, 80–82]. In low-risk cohorts receiving KV-based IORT, the reports are stating satisfactory cosmetic results (excellent/good: 89–97%) [83–86].

The interpretation of randomized trials comparing PBI and WBI is often difficult due to several factors changed in the PBI arms. Investigators changed not only the treated breast volume, but also the fractionation schedule, number of daily treatments and radiation technique which interferes with the genuine study query. Only two trials used the same technique and fractionation schedule and randomized only to the treated breast volume [35, 36]. Both of them reported favourable point estimates for all evaluated toxicities, patient-reported outcomes, cosmetic results and QoL analyses.

Data from multiple randomized trials suggest that the difference in oncologic endpoints between partial- and whole breast radiation therapy is very limited [21, 22, 48, 71]. This observation strengthens the necessity of an analysis of adverse events as well as quality of life. Comparative research suggests that patients’ priorities when weighing side effects and QoL compared to oncologic cure are similarly important [87]. In addition to equal recurrence and survival outcomes, Shah and colleagues demonstrated that multiple PBI regimes are cost effective, both per cost-effectiveness ratio analysis and cost per quality adjusted life year compared to hypofractionated WBI [88, 89].

## Limitations

A limitation of our study is the use of published data rather than individual patient data which would be generally preferable. However, meta-analyses of aggregated patient data have been also shown to provide valuable conclusions [90]. Pooling of different toxicity scales can introduce bias in the analysis [91]. Yet, a good correlation between the LENT-SOMA and the RTOG/EORTC toxicity scales has been reported [92]. The strategy of using the last available time point during follow-up reduced the number of patients, and ensured the detection of possible toxicities. Further, the prevalence of breast hardness, pain, oversensitivity, edema, and skin changes is reduced over follow-up whereas breast shrinkage increased [52]. The use of conventionally fractionated instead of hypofractionated radiation therapy (HFX) in the standard arm of most trials introduces a bias towards PBI, especially regarding skin toxicity. The pooled analysis of the UK START trials, a Cochrane meta-analysis as well as other randomized trials demonstrated reduction in the adverse events as well as improved QoL and cosmetic results with hypofractionated WBI [52, 93–97].

A noticeable strength of our analysis is the use of multiple toxicity endpoints, separated by grading and different follow-up intervals as well as the differentiation by PBI technique. A follow-up period with a median of 8.6 years should be adequate to capture the majority of adverse events.

## Conclusion

A reduction of the breast volume treated by adjuvant radiotherapy reduces acute skin toxicity and improves breast symptom-related quality of life. Twice-daily fractionation leads to higher fibrosis and worse cosmesis.

## Figures and Tables

**Fig. 8:**
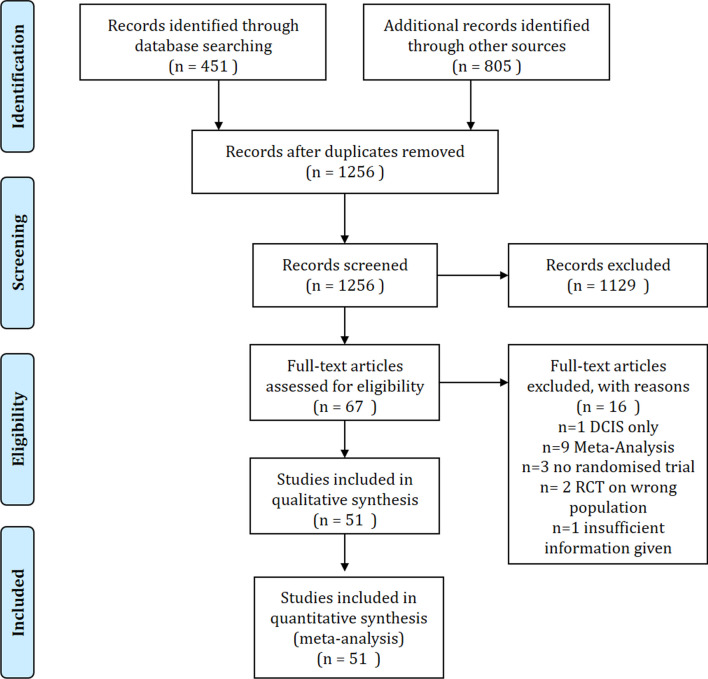
Consort diagram of the literature search according to the PRISMA guideline

**Fig. 9:**
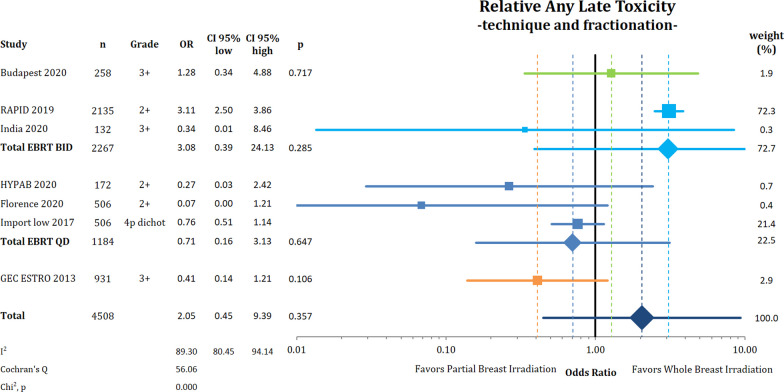
Comparison of any late toxicity by technique using odds ratios with the respective 95% confidence intervals between partial breast radiotherapy and whole breast radiotherapy. The green line represents the comparison of EBRT BID/BT to WBI. The orange line represents the comparison of BT to WBI

**Fig. 10:**
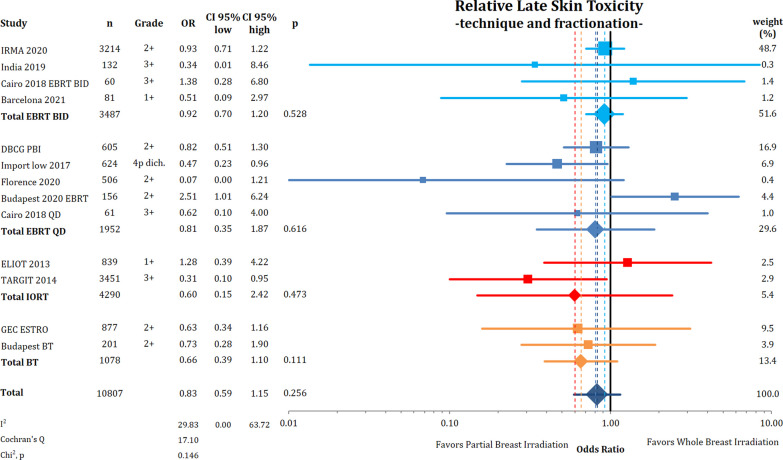
Comparison of late skin toxicity by technique using odds ratios with the respective 95% confidence intervals between partial breast radiotherapy and whole breast radiotherapy

**Fig. 11:**
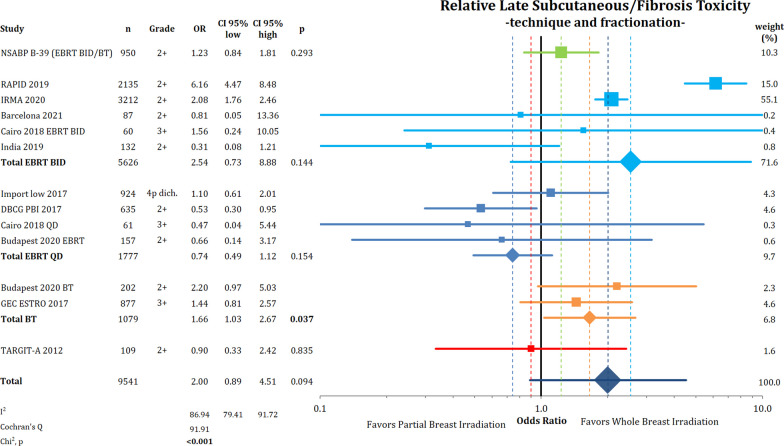
Comparison of late subcutaneous tissue/fibrosis toxicity by technique using odds ratios with the respective 95% confidence intervals between partial breast radiotherapy and whole breast radiotherapy. The green line represents the comparison of EBRT BID/BT to WBI. The red line represents the comparison of BT–WBI. The green line represents the comparison of EBRT BID/BT to WBI. The orange line represents the comparison of BT to WBI

**Fig. 12:**
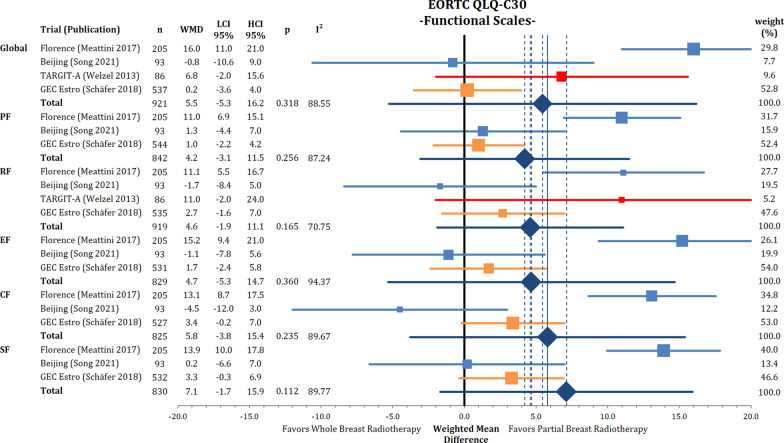
Comparison of QLQ-C30 functional scores between partial breast irradiation and whole breast irradiation in different subdomains using weighted mean differences. Higher functional scores represent better QoL. PF = physical functioning, RF = role functioning, EF = emotional functioning, CF = cognitive functioning, SF = social functioning

**Fig. 13:**
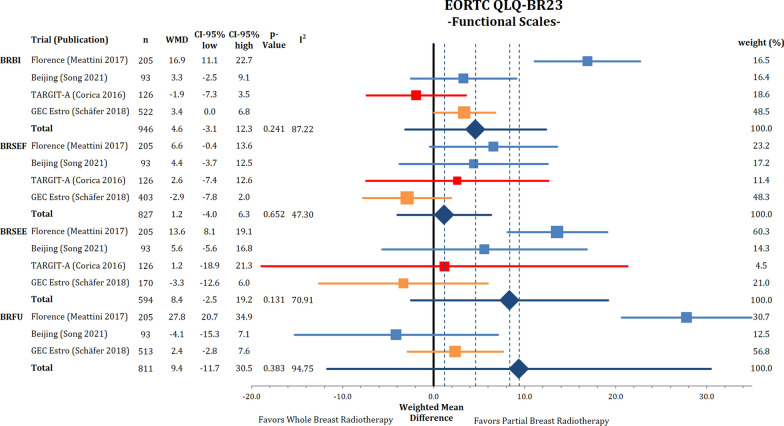
Comparison of QLQ-BR23 functional scores between partial breast irradiation and whole breast irradiation in different subdomains using weighted mean differences. Higher functional scores represent better QoL. BRBI = body image, BRSEF = sexual functioning, BRSEE = sexual enjoyment, BRFU = future perspective

## Appendix

See Figs. 8, 9, 10, 11, 12, 13 and Tables 1 and 2.

**Table 1:** .

Synonym	y trial	FU	N total	Med. Age	Stat Setting	Prim. EP	Pop	Strat	PBI Technique	PBI Dose	WBI Dose	G3	DCIS	N+	HR+	Her2 neu	CTx	ET	Boost
NSABP B-39	2005– 2013	10.2	4216	54	Equiv	IBTR	IBC or DCIS; T < 3 cm, ≤ N1; R0; > 18y	Stage, Menopausal, ER, CTx	3DCRT, single- and multicath. BT	34/3.4; 38.5/3.85 10 × in 5-8d	50/2; 50.4/1.8; opt. Boost	26%	24%	10%	81%	n.r	29%	n.r	80%
Budapest	1998– 2004	17	258	Mean 59	Noninf	LR	IBC; T < 2 cm; N0; R0; G1-2; unifocal	None	multicath. BT 3DCRT e-	BT:36.4/5.2 BID; e-:50/2 QD	50/2 + opt. 16/2	0.0%	0%	3% N1mic	88%	n.r	3%	99%	0.8%
RAPID	02/2006– 07/2011	8.6	2135	61	Noninf	IBTR	IBC or DCIS; T < 3 cm;R0; N0; > 40y; unifocal	Age > < 50; Histology, T > < 1.5 cm; ER, centre	3DCRT IMRT	38.5/3.85 BID in 5-8d	50/2; 42.5/2.66 + opt. Boost	16%	18%	0%	84%	6%	13%	55%	21%
IRMA	2007– 2019	5	3309	65	Noninf	IBTR	IDC; T < 2.5 cm; R0; > 45y; unifocal	Centre, timing	3DCRT	38.5/3.85 BID	50/2; 50.4/1.8; 45/2.5 + opt. Boost 10–16/2	n.r	0%	7%	95%	n.r	6%	74%	n.r
Barcelona	n.r	10.3	102	Mean 69	Noninf	IBTR	IBC; T < 3 cm;R0; N0; > 60y; unifocal; G 1–2	n.r	3DCRT	37.5/3.75 BID	48/2 + opt. Boost 10/2	0.0%	0%	0%	98%	1%	3%	57%	n.r
India	06/2011–12/2015	5	133	Mean 50	Noninf	LR	IDC, T < 4 cm, cN0, pN0-1, R0	None	3DCRT	34/3.4 BID	40/2.5 + opt 10–16/2	23%	0%	12%	69%	11%	65%	74%	56%
Cairo	n.r	2	91	50	n.r	IBTR	IBC; T < 3 cm;R0; N0; > 40y;unifocal; G1-3	n.r	3DCRT	38,5/3,85 QD 38.5/3.85 BID	50/2 + opt. Boost	n.r	0%	0%	80%	n.r	19%	n.r	n.r
Florence	03/2005– 06/2013	10.7	520	n.r	noninf	IBTR	IBC or DCIS; T < 2.5 cm; > 40y; BCS +	None	IMRT	30/6 q.o.d	50/2 + opt. Boost 10/2	11.4%	11%	10%	96%	4%	7%	62%	n.r
Import low	05/2007– 10/2010	6	1343	62	noninf	IBTR	IDC; T < 3 cm; > 50y; pN0-1	Centre	3DCRT	40/2.67 QD	40/2.67	9.7%	0%	3%	95%	4%	5%	80%	n.r
DBCG PBI	2009– 2016	7.6	882	66	noninf	Breast Induration 3y	IBC, T1, R0, > 60y, G1-2, HER2-, pN0	Centre, ET	3DCRT	40/2.67 QD	40/2,67	< 1.0%	0%	0%	100%	0%	n.r	80%	n.r
HYPAB	01/2015–01/2018	3	172	64	n.r	Cosmetic outcome	Postmen. T1-2 pN0, unicentric, R0 (5 mm), No EIC	n.r	VMAT	30/6 alternate days	40.5/2.7 48/3.2	3%	n.r	0%	100%	n.r	n.r	97%	100%
Beijing	06/2017– 01/2019	1	115	54	noninf	Grade II + Tox	45-75y, G1-2, T < 3 cm, pN0, unifocal, ER/PR + , R0 (2 mm)	None	IMRT	40/4 QD	43.5/2.9 Opt. SIB 49.5/3.3	0%	n.r	0%	100%	7%	21%	n.r	3%
ELIOT	11/2000– 12/2007	12.4	1305	n.r	noninf	IBTR	IBC; T < 2.5 cm; R0; 48-75y; unifocal	T < 1–1.4 > cm	IORT e-	21/21	50/2 + opt. Boost 10/2	20.9%	0%	27%	91%	3%	8%	88%	n.r
TARGIT-A prepathology	03/2000– 06/2012	8.6	2298	Mean 63	noninf	LRFS	IDC; T < 2.5 cm; R0; > 45y; unifocal	Centre, timing	IORT x	20/20	n.r	20%	0%	~ 21%	90%	15%	21%	81%	38%
TARGIT-A postpathology	03/2000– 06/2012	9	1153	Mean 63	noninf	LRFS	IDC; T < 2.5 cm; R0; > 45y; unifocal	Centre, timing	IORT x	20/20	n.r	6%	3%	5%	98%	6%	4%	87%	n.r
GEC Estro	04/2004– 07/2009	10.4	1328	62	noninf	IBTR	IBC or DCIS; T < 3 cm; R0; N0; > 40y; BCS +	Centre, Menopausal, stage	multicath. BT	32/4; 30.3/4.3 or PDR	50/2; 50.4/1.8; Boost opt	8.3%	5%	6%	95%	n.r	11%	90%	98%

**Table 2:** .

Trial	Additional publications	Cosmetic evaluation	Quality of Life	Any acute	Acute skin	Acute lung	Acute breast pain	Any late	Late skin	Late subcutaneous/tissue	Late telangiectasia	Late breast edema	Late breast pain	Late chest- wall pain	Late Fat Necrosis	Late lung
NSABP B-39	Vicini 2019 [98] White 2019 [25] Vicini 2019 [50]	4p GCS: 3y (MP) [25] 4p GCS: 3y (Pat) [25]	BCTOS, Pain Score, SF-36 [99]	n.r	n.r	n.r	n.r	n.r	n.r	n.r	n.r	n.r	n.r	n.r	n.r	n.r
Budapest	Polgar 2004 [100] Polgar 2007 [101] Lövey 2007 [102] Polgar 2013 [103] Polgar 2014 [104] Polgar 2020 [27]	4p: 5y, 10y, 20y (MP) [27, 101, 104]	n.r	n.r	n.r	n.r	n.r	RTOG/EORTC G3 + [104]	RTOG/EORTC G1 + ,G2 + ,G3 + [27]	RTOG/EORTC G1 + ,G2 + ,G3 + [27]	RTOG/EORTC G3 + [104]	n.r	n.r	n.r	G1 + ,G2 + ,G3 + ,G4 + [102]	n.r
RAPID	Olivotto 2013 [105] Peterson 2015 [53] Whelan 2019 [106] Whelan 2019 [26]	4p: 3y,5y,7y (Nurse) [26] 4p: 3y,5y,7y (Pat) [26]	n.r	CTCAE V3: G2 + ,G3 + [26]	CTCAE V3: G2 + ,G3 + [26]	CTCAE V3: G2 + ,G3 + [26]	CTCAE V3: G2 + ,G3 + [26]	CTCAE V3: G2 + ,G3 + [26]	n.r	CTCAE V3: G2 + ,G3 + [26]	CTCAE V3: G2 + ,G3 + [26]	n.r	CTCAE V3: G2 + ,G3 + [26]	CTCAE V3: G2 + ,G3 + [26]	CTCAE V3: G2 + ,G3 + [26]	n.r
IRMA	Meduri 2017 [33] Meduri 2020 [107] Meduri 2023 [108]	4p: 1y,3y,5y (MP) [107] 4p: 1y,3y,5y (Pat) [107]	n.r	n.r	RTOG/EORTC G1 + ,G2 + ,G3 + [107, 108]	n.r	n.r	n.r	RTOG/EORTC G1 + ,G2 + ,G3 + [107, 108]	RTOG/EORTC G1 + ,G2 + ,G3 + [107, 108]	n.r	n.r	n.r	RTOG/EORTC Bone G1 + ,G2 + ,G3 + [107]	n.r	RTOG/EORTC G1 + ,G2 + ,G3 + [107]
Barcelona	Rodriguez 2013 [30] Li 2021 [109]	4p: 1y,3y,5y,10y (MP) [30, 109]	n.r		RTOG/EORTC G1 + ,G2 + .G3 + [30]	RTOG/EORTC G2 + [30]	n.r	RTOG/EORTC G1 + [109]	RTOG/EORTC G1 + [109]	RTOG/EORTC G1 + , G2 + [109]	n.r	n.r	RTOG/EORTC G1 + [109]	n.r	n.r	n.r
India	Yadav 2019 [51]	4p: 5y (MP) [51]	n.r	n.r	RTOG/EORTC G1 + ,G2 + ,G3 + [51]	n.r	n.r	RTOG/EORTC G3 + [51]	RTOG/EORTC G3 + [51]	RTOG/EORTC induration G1 + ,G2 + [51]	n.r	RTOG/EORTC G1 + ,G2 + [51]	n.r	n.r	RTOG/EORTC G1 + [51]	n.r
Cairo	Boutrus 2018 [24]	4p: 6 m,1y [24]	n.r		RTOG/EORTC G3 + [24]	RTOG/EORTC G1 + [24]	n.r	n.r	RTOG/EORTC G3 + [24]	RTOG/EORTC G3 + [24]	RTOG/EORTC G1 + [24]	n.r	n.r	n.r	n.r	n.r
Florence	Livi 2010 [77] Livi 2015 [110] Meattini 2017 [111] Meattini 2020 [112] Meattini 2020 [32]	4p: 5y,10y (MP) [32, 110] 4p: 5y,10y (Pat) [32, 110]	EORTC QLQ-C30 [111] EORTC QLQ-BR23 [111]	RTOG/EORTC: G1 + ,G2 + ,G3 + [32]	RTOG/EORTC: G1 + ,G2 + ,G3 + [112]	n.r	n.r	RTOG/EORTC: G1 + ,G2 + [32]	RTOG/EORTC: G1 + ,G2 + [112]	n.r	n.r	n.r	n.r	n.r	n.r	n.r
Import low	Coles 2017 [35] Bhattacharya 2019 [52] Bhattacharya 2019 [113] Bhattacharya 2019 [114]	3p PA: 2y,5y (MP) [35] 4p: 5y (Pat: Breast appearance changed) [35]	4p dich. EORTC QLQ-C30 [35] 4p dich. EORTC QLQ-BR23 [35]	n.r	n.r	n.r	n.r	4p “Worst normal-tissue effect” [35] Different scale	4p “Skin problems in breast” (Pat) [35] Different scale	4p breast induration (index) [35] Different scale	4p teleangiectasia [35] Different scale	4p breast edema [35] Different scale	4p breast pain (pain) [35] Different scale	Symptomatic rib fracture reported [35] different scale	n.r	Symptomatic lung fibrosis reported [35] different scale
DBCG PBI	Offersen 2017 [29] Offersen 2022 [36]	4p GCS: 3y 5y (MP) 4p: 3y 5y (Pat satisfaction) [29, 36]	n.r	n.r	n.r	n.r	n.r	n.r	Scar appearance G2 + [36]	4p Breast Induration G2 + [36]	4p teleangiectasia G2 + [36]	4p edema G2 + [36]	4p pain G2 + [36]	n.r	n.r	n.r
HYPAB	Franceschini 2020 [115]	4p GCS: 6 m (MP) [115]	n.r	CTCAE V4: G1 + , G2 + , G3 + [115]	CTCAE V4 G1 + , G2 + , G3 + [115]	n.r	n.r	CTCAE V4: G1 + , G2 + , G3 + [115]	n.r	n.r	n.r	n.r	n.r	n.r	CTCAE V4 G3 + [115]	
Beijing	Song 2021 [116]	n.r	EORTC QLQ-C30 [116] EORTC QLQ-BR23 [116]	n.r	n.r	n.r	n.r	n.r	n.r	n.r	n.r	n.r	n.r	n.r	n.r	
ELIOT	Veronesi 2013 [31] Orecchia 2021 [117]	n.r	n.r	n.r	LENT SOMA G1 + ,G3 + ,G4 + [31]	n.r	n.r	n.r	LENT SOMA G1 + [31]	n.r	n.r	n.r	n.r	n.r	LENT SOMA G1 + [31]	LENT SOMA G1 + ,G2 + ,G3 + [31]
TARGIT-A prepathology	Vaidya 2010 [118] Andersen 2012 [119] Sperk 2012 [120] Welzel 2013 [60] Keshtgar 2013 [121] Vaidya 2014 [122] Vaidya 2016 [123] Corica 2016 [124] Corica 2018 [34] Vaidya 2020 [125] Vaidya 2020 [126]	4p: 1y,2y,3y,4y,5y (Nurse) [34] 4p: 1y,2y,3y,4y,5y (Pat) [34]	EORTC QLQ-C30 [60, 124] EORTC QLQ-BR23 [60, 124]	RTOG G3 + [123]	RTOG G3 + [123]	n.r	n.r	n.r	RTOG/EORTC G3 + [123]	LENT SOMA G2 + ,G3 + Cum inc (3y) [120]	LENT SOMA G1 + Cum inc (3y) [120]	LENT SOMA G2 + [120]	Any breast pain G1 + [119] LENT SOMA G2 + Cum inc (3y) [120]- Different scales	n.r	n.r	n.r
TARGIT-A postpathology																
GEC ESTRO	Ott 2016 [127] Strnad 2016 [128] Polgar 2017 [129] Schäfer 2018 [28] Strnad 2023 [130]	4p: 1y,3y,5y (MP) [129] 4p: 1y,3y,5y (Pat) [129]	EORTC QLQ-C30 [28] EORTC QLQ-BR23 [28]	CTCAE V3 G3 + [127]	CTCAE V3 G1 + ,G2 + ,G3 + [127]	n.r	CTCAE V3 G1 + ,G2 + [127]	RTOG/EORTC G1 + ,G3 + [130]	RTOG/EORTC Skin G1 + ,G2 + ,G3 + [129, 130]	RTOG/EORTC subcutaneous tissue G1 + ,G2 + ,G3 + [129, 130]	LENT SOMA teleangiectasia G1 + ,G2 + ,G3 + [129, 130]	n.r	LENT SOMA pain G1 + ,G2 + ,G3 + [129]	n.r	LENT SOMA fat necrosis G1 + ,G2 + ,G3 + [129]	n.r

## Abbreviations

ASCO: American Society for Clinical Oncology
ASTRO: American Society for Radiation Oncology
BCS: Breast conserving surgery
BCTOS: Breast Cancer Treatment Outcome Scale
BED: Biologically effective dose
BID: Twice a day radiation therapy
BMI: Body mass index
BT: Brachytherapy
CFX: Conventionally fractionated radiation therapy
CI: Confidence interval
CTCAE: Common Terminology Criteria for Adverse Events
DBCG: Danish Breast Cancer Cooperative Group
EBRT: External beam radiation therapy
ESTRO: European Society for Radiation Oncology
ESMO: European Society for Medical Oncology
EORTC: European Organization for Research and Treatment of Cancer
HFX: Hypofractionated radiation therapy
IMRT: Intensity-modulated radiation therapy
KV: Kilovoltage
ivhet: Inverse variance heterogeneity model
LENT-SOMA: Late effects of normal tissues-subjective objective management analytic
OR: Odds ratio
PBI: Partial breast irradiation
PRISMA: Preferred Reporting Items for Systematic reviews and Meta-analyses
QoL: Quality of life
QD: Once a day radiation therapy
QLQ: Quality of life questionnaire
QLQ-BR23: Questionnaire for measuring the quality of life in patients with breast cancerQLQ-C30: quality of life of cancer patient’s questionnaire
RAPID trial: Randomized Trial of Accelerated Partial Breast Irradiation
RTOG: Radiation Therapy Oncology Group
START: Standardization of breast radiotherapy
TARGIT: Targeted intraoperative radiotherapy
UK: United Kingdom
WBI: Whole breast irradiation
WMD: Weighted mean differences
